# Polymicrobial endophthalmitis: prevalence, causative organisms, and visual outcomes

**DOI:** 10.1186/1869-5760-3-6

**Published:** 2013-01-07

**Authors:** Animesh Jindal, Mayur R Moreker, Avinash Pathengay, Manav Khera, Subhadra Jalali, Ajit Majji, Annie Mathai, Savitri Sharma, Taraprasad Das, Harry W Flynn

**Affiliations:** 1 LV Prasad Eye Institute, GMRV Campus, Visakhapatnam, India; 2 LV Prasad Eye Institute, KAR Campus, Hyderabad, India; 3 Bhubaneswar Eye Institute, LV Prasad Eye Institute, Bhubaneswar, India; 4 Department of Ophthalmology, Bascom Palmer Eye Institute, Miller School of Medicine, University of Miami, Miami, FL, USA

**Keywords:** Polymicrobial, Endophthalmitis, Antibiotic susceptibilities

## Abstract

**Background:**

The purpose of the present study is to evaluate the prevalence, causative organisms, and visual acuity outcome in patients with culture-proven polymicrobial endophthalmitis. The method used in this study is the non-comparative, consecutive case series using a retrospective analysis of patients diagnosed with polymicrobial endophthalmitis for the period 2000 to 2010.

**Results:**

Polymicrobial endophthalmitis was identified in 43/1,107 (3.88%) patients. Forty-two patients had two isolates, and one patient had grown three isolates, yielding a total of 87 isolates. Gram-positive cocci were the most common isolate (*n* = 53; 60.9%) including *Staphylococcus epidermidis* (*n* = 14/53; 16.1%) and *Streptococcus pneumoniae* (*n* = 13/53; 13.8%). The etiologies included posttraumatic (*n* = 31/43; 72.1%) and postoperative (*n* = 9/43; 20.9%) endophthalmitis. Antibiotic susceptibilities among Gram-positive bacteria were vancomycin (100%) and chloramphenicol (96%). Susceptibilities among Gram-negative bacteria were ciprofloxacin (86.4%) and ofloxacin (81.2%). A maximum number of secondary interventions were done in traumatic cases (38.7%) and cases having coinfection with Gram-negative bacteria and fungus (66.7%). Visual acuity (VA) < 20/200 was more frequently observed in posttraumatic cases (*n* = 27/31; 87.1%) as compared with postoperative cases (*n* = 4/9; 44.4%). Of the 43 patients, only 9 patients (20.9%) achieved a VA ≥ 20/200 on final follow-up. Four out of twelve patients (33.3%), with fungus as one of the isolates, had a VA ≥ 20/200.

**Conclusions:**

Although polymicrobial infection in endophthalmitis is uncommon, it is generally associated with poor visual acuity outcomes especially in eyes with open-globe injuries. Coinfection with Gram-negative bacteria or fungi was associated with most unfavorable visual outcome.

## Background

Endophthalmitis is one of the most vision-threatening ocular complications following intraocular surgeries and open-globe injuries. The incidence of polymicrobial infection in the report of the Endophthalmitis Vitrectomy Study (EVS) Group was 9.3% [1]. The incidence of polymicrobial endophthalmitis after open-globe injuries has been reported from 5.3% to 47.6% [2-6], while it has been reported to be 0.0% to 17% in various postoperative endophthalmitis series [6-12]. There are limited published series reporting the etiology and outcome of polymicrobial endophthalmitis [12]. No large series are available in the literature on etiology and visual acuity (VA) outcomes of polymicrobial endophthalmitis cases.

The purpose of the present study is to evaluate the prevalence, causative organisms, and visual acuity outcomes in patients with culture-proven polymicrobial endophthalmitis in a teaching hospital.

## Results and discussion

### Results

Polymicrobial endophthalmitis was seen in 43 (3.88%) out of 1,107 culture-proven endophthalmitis patients. There were 31 male patients as compared to 12 female patients with polymicrobial infection. Forty-two patients had grown two isolates and one patient had grown three isolates, yielding a total of 87 isolates (Table 1).

**Table 1:** Clinical profile of patients with polymicrobial endophthalmitis

**SR number**	**Age**/**gender**	**Setting**	**Visual acuity** (**presenting**)	**Primary intervention**	**Secondary intervention** (**Y**/**N**)	**Visual acuity** (**final**)	**Organisms**
1	7 years/F	Trauma	PR inacc	PPV + IOAB	N	No PL	*Staphylococcus epidermidis*, *Streptococcus pneumoniae*
2	5 years/M	Trauma	PR inacc	STR + PPV + IOAB	N	CF 2 m	*Alpha hemolytic Streptococcus*, *Haemophilus influenzae*
3	60 years/M	Cataract surgery	20/200	Vit Bx + IOAB	N	20/400	*Bacillus*, *Corynebacterium*
4	6 years/F	Trauma	PR inacc	STR + PPV + IOAB	N	No PL	*Alpha hemolytic Streptococcus*, *Acromonas*
5	8 years/F	Trauma	PR inacc	STR + PPV + IOAB	N	No PL	*Bacillus*, *S*. *pneumoniae*
6	35 years/F	Trauma	PR inacc	IOFB removal + PPV + IOAB	N	CF 1/2 m	*Aspergillus* sp., *Bacillus*
7	45 years/M	Trauma	PR inacc	STR + PPV + IOAB	N	No PL	*S*. *epidermidis*, *Bacillus*
8	15 years/M	Trauma	PR acc	IOFB removal + PPV + IOAB	N	20/60	*Alpha hemolytic Streptococcus*, *Clostridium*
9	64 years/M	Penetrating keratoplasty	20/1200	Vit Bx + IOAB	N	20/160	*S*. *epidermidis*, *S*. *pneumoniae*
10	36 years/M	Trabeculectomy	PR inacc	PPV + IOAB	N	20/40	*S*. *pneumoniae*, *Fusarium*
11	73 years/M	Cataract surgery	PR acc	PPV + IOAB	N	PR inacc	*S*. *epidermidis*, *Aspergillus*
12	8 years/M	Trauma	PR inacc	IOFB removal + PPV + IOAB	N	No PL	*S*. *epidermidis*, *Alpha hemolytic Streptococcus*
13	39 years/F	Trauma	PR inacc	STR + PPV + IOAB	N	PR inacc	*Chrysomonads*, *Fusarium*
14	23 years/M	Trauma	PR acc	PPV + IOAB	N	20/400	*S*. *epidermidis*, *Aspergillus*
15	4 years/M	Trauma	PR inacc	STR + PPV + IOAB	N	PR acc	*S*. *epidermidis*, *Aspergillus*
16	16 years/M	Trauma	PR inacc	IOFB removal + PPV + IOAB	N	PR inacc	*S*. *epidermidis*, *S*. *pneumoniae*
17	71 years/M	Trabeculectomy	PR acc	STR + PPV + IOAB	N	20/50	*Moraxella*, *S*. *epidermidis*
18	45 years/M	Cataract surgery	20/25	Vit Bx + IOAB	N	20/25	*S*. *epidermidis*, *Aspergillus*
19	18 years/M	Trauma	PR acc	PPV + IOAB	N	20/1200	*S*. *epidermidis*, *Aspergillus*
20	38 years/M	Trauma	PR acc	PPV + IOAB	N	20/40	*S*. *epidermidis*, *Clostridium*
21	60 years/M	Cataract surgery	PR acc	PPV + IOAB	N	PR inacc	*Corynebacterium*, *Aspergillus*
22	38 years/M	Trauma	PR acc	STR + PPV + IOAB	Y	PR acc	*Bacillus*, *Alpha hemolytic Streptococcus*
23	14 years/M	Endogenous	Pr acc	PPL + PPV + IOAB	N	No PL	*S*. *pneumoniae*, *S*. *aureus*
24	4 years/F	Trauma	PR acc	PPV + IOAB	N	No PL	*S*. *pneumoniae*, *H*. *influenzae*
25	50 years/M	Trauma	PR inacc	ECCE + PPV + IOAB	Y	No PL	*S*. *pneumoniae*, *Pseudomonas*
26	57 years/M	Penetrating keratoplasty	PR acc	PPV + IOAB	N	CF-1/2m	*S*. *pneumoniae*, *H*. *influenzae*
27	7 years/F	Trauma	PR acc	STR + PPV + IOAB	Y	No PL	*S*. *pneumoniae*, *E*. *coli*
28	7 years/F	Trauma	PR acc	PPL + PPV + IOAB	N	No PL	*S*. *pneumoniae*, *Neisseria*
29	3 years/M	Trauma	PR acc	PPV + IOAB	N	No PL	*S*. *pneumoniae*, *Pseudomonas*
30	8 years/M	Trauma	PR acc	PPL + PPV + IOAB	Y	No PL	*S*. *pneumoniae*, *H*. *influenzae*
31	11 years/M	Trauma	PR inaac	CTR + PPL + PPV + IOAB	N	No PL	*Bacillus*, *Enterococcus*, *Pantoea*
32	6 years/M	Trauma	PR acc	STR + PPL + PPV + IOAB + IOFB Removal	Y	PL	*Enterococcus*, *Stenotrophomonas*
33	3 years/F	Trauma	No PL	STR + PPL + PPV + IOAB	N	No PL	*Paenibacillus thiaminolyticus*, *Enterobacter*
34	1 month/M	Endogenous	PL	Vit Bx + IOAB	N	No PL	*S*. *pneumoniae*, *Pseudomonas*
35	4 years/M	Trauma	PR acc	CTR + PPL + PPV + IOAB	Y	HM+	*Streptococci*, *Fusarium*
36	57 years/F	Cataract surgery	20/40	Vit Bx + IOAB	Y	20/20	*Pseudomonas*, *Aspergillus*
37	3 years/M	Trauma	PL+	CTR + PPL + PPV + IOAB	Y	CF-2m	*S*. *epidermidis*, *Corynebacterium*
38	14 years/M	Trauma	PR inacc	PPL + PPV + IOAB	Y	20/200	*Streptococcus mitis*, *Fusarium*
39	5 years/M	Trauma	PR acc	PPV + IOAB	Y	PL	*Streptococci*, *Pseudomonas*
40	31 years/M	Endogenous	PR acc	PPV + IOAB	Y	HM+	*S*. *epidermidis*, *E*. *coli*
41	18 years/F	Trauma	PL	PPL + PPV + IOAB + Ampho B	Y	20/25	*Pseudomonas*, *S pneumonia*
42	19 years/M	Trauma	HM+	CTR + PPL + PPV + IOAB + Ampho B	Y	PL	*Streptococci*, *Pseudomonas*
43	34 years/F	Trauma	PL	PPV + IOAB	Y	PL	*Corynebacterium*, *Aspergillus*

*PR* acc, projection of rays accurate; *PR* inacc, projection of rays inaccurate; *HM+*, hand motion close to face present; *PL*, perception of light; *PPL*, pars plana lensectomy; *PPV*, pars plana vitrectomy; *Vit Bx*, vitreous biopsy; *IOAB*, intraocular antibiotics; *IOFB*, intraocular foreign body; *CTR*, corneal tear repair; *STR*, scleral tear repair; *Ampho B*, intravitreal amphotericin B.

### Clinical presentation

Overall, the presenting visual acuity was light perception in 38 patients and was greater than or equal to hand motion in 5 patients. Thirty-eight patients underwent core vitrectomy, and five patients underwent vitreous biopsy as the first intervention. In all the patients, the organisms were isolated and identified from the first vitreous sample, collected during the first procedure (vitreous biopsy/vitrectomy). Gram-positive organisms were the most common isolates (*n* = 53; 60.9%), followed by Gram-negative organisms (*n* = 22; 25.3%) and fungi (*n* = 12; 13.8%). *Staphylococcus epidermidis* (*n* = 14; 16.1%) and *Streptococcus pneumoniae* (*n* = 13; 14.9%) were the most common organisms. The endophthalmitis categories were open-globe injury (31/43; 72.1%), postoperative endophthalmitis (9/43; 20.9%), and endogenous endophthalmitis (3/43; 6.9%) (Table 2).

**Table 2:** Comparison of secondary interventions and visual outcome among different etiologies of polymicrobial endophthalmitis

	**Trauma**	**Postoperative**	**Endogenous**
Number of patients	31	9	3
Undergoing secondary intervention	12 (38.7%)	1 (11.1%)	1 (33.3%)
Unfavorable visual outcome (VA < 20/200)	27 (87.1%)	4 (44.4%)	3 (100%)

### Antibiotic susceptibilities

Gram-positive bacteria were most sensitive to vancomycin (100%) and chloramphenicol (96%). Gram-negative bacteria were most sensitive to ciprofloxacin (86.4%) and ofloxacin (81.2%) (Table 3).

**Table 3:** Antibiotic susceptibility of isolates in polymicrobial endophthalmitis

**Antibiotic**	**Sensitivity of gram**-**positive bacteria(%)**	**Sensitivity of gram**-**negative bacteria(%)**
Amikacin	57.1	61.9
Cefazolin	81.1	33.3
Ceftazidime	61.1	63.1
Ciprofloxacin	82.7	86.4
Chloramphenicol	96	72.7
Gentamycin	74	63.6
Gatifloxacin	82.6	80
Ofloxacin	67.7	81.2
Vancomycin	100	45.4
Moxifloxacin	88.2	70

### Secondary intervention

Twelve out of 31 patients (38.7%) having endophthalmitis following open-globe injury had secondary intervention as compared to postoperative (1/9; 11.1%) and endogenous (1/3; 33.3%) endophthalmitis. The number of patients requiring additional procedures was maximum for combination of Gram-negative and fungus (66.7%) followed by Gram-positive and Gram-negative combination (42.1%) (Tables 2 and 4).

**Table 4:** Comparison of secondary interventions and visual outcome among different microbes in polymicrobial endophthalmitis

	**Gram**-**positive and gram**-**negative combination**	**Gram**-**positive and gram**-**negative combination**	**Gram**-**positive and fungus combination**	**Gram negative and fungus combination**
No. of patients	12	19	9	3
Undergoing secondary intervention	2 (16.7%)	8 (42.1%)	2 (22.2%)	2 (66.7%)
Unfavorable visual outcome (VA < 20/200)	9 (75%)	17 (89.5%)	6 (66.7%)	2 (66.7%)

### Visual outcomes

An unfavorable visual outcome (VA < 20/200) resulted in 27 out of 31 patients (87.1%) having posttraumatic endophthalmitis and four out of nine (44.4%) patients having postoperative endophthalmitis. Combination of Gram-positive and Gram-negative organisms had the worst visual prognosis with only 10.5% patients having final VA ≥ 20/200. Out of the 43 patients, only 9 (20.9%) patients had a best corrected VA ≥ 20/200 at final follow-up. Eight out of 12 patients (66.67%) having fungi as one of the infecting organisms had a VA < 20/200 at final follow-up visit.

## Discussion

Polymicrobial eye infections present a challenge not only in identifying two or more microorganisms, but also in instituting appropriate antimicrobial therapy. In the current study and in large series, polymicrobial infections seem to occur more frequently in open-globe injuries, underscoring the non-sterile conditions under which ocular trauma occurs. Polymicrobial infections have been reported following advanced keratitis, infected scleral buckles, and dacryocystitis [13-15]. In a retrospective study from North India, Gupta et al. reported polymicrobial endophthalmitis in 8 eyes out of 47 culture-positive postoperative endophthalmitis eyes (17%) [11]. Pijl et al. from Netherlands found polymicrobial infection in 4 out of 166 culture-positive cases (2.4%) of postoperative endophthalmitis [12]. Anand et al., in a series of 170 culture-proven postoperative endophthalmitis, reported 3 cases (1.8%) of polymicrobial endophthalmitis [16]. Vedantham et al., reported 3 (7.7%) polymicrobial endophthalmitis cases in a series of 39 posttraumatic patients [17]. In the author's previous published report for the period 1991 to 1997, polymicrobial infections were identified in 12.5% (14 of 112) culture-positive postoperative cases, while it was present in 20.4% (23 of 113) culture-positive posttraumatic cases, with three trimicrobial cases [2,9]. In the current series from 2000 to 2010, less prevalence of polymicrobial infection (3.88%) was observed compared with the earlier report. The cause for this decrease in prevalence of polymicrobial infection is uncertain.

Gram-positive bacteria (*S*. *epidermidis* and *S*. *pneumoniae*) are the most common isolates from polymicrobial endophthalmitis in the current series, contrasting with the previous series where Gram-negative bacteria and fungus were most common isolates.

Considering the very short half-life of fluoroquinolones but good penetration in vitreous cavity, this class of antibiotics can be considered for per-oral therapy, and vancomycin can be considered for intravitreal injections. Repeat intravitreal antibiotics if needed should be based on culture sensitivity report.

Patients with posttraumatic endophthalmitis had poorer visual outcomes when compared with postoperative endophthalmitis, which is consistent with our previous series and previous reported literature [2,7-9]. Five out of nine postoperative patients (55.5%) having polymicrobial infection had favorable visual outcome as compared with only 4 patients out of 31 (12.9%) having posttraumatic endophthalmitis. The number of patients undergoing additional procedure was also more for posttraumatic cause (38.7%) as compared with postoperative cause (11.1%). We had three patients with endogenous source of infection who had polymicrobial infection, and all three had unfavorable visual outcome.

Although prevalence of fungus in our series has decreased, given the unsterile conditions under which traumatic endophthalmitis occurs and high probability of fungal contamination, intravitreal antifungal agents should be considered along with antibiotics, but decision should be individualized based on history and clinical examination. Limitations of the current study include the retrospective nature and lack of a definitive prospective treatment protocol.

## Conclusions

Polymicrobial infections in endophthalmitis are uncommon and are often associated with trauma. Coinfection with Gram-negative bacteria or fungi may be associated with the most unfavorable visual acuity outcomes.

## Methods

Approval was obtained from the local Institutional Review Board. All the patients who were diagnosed with endophthalmitis during the period 2000 to 2010 were analyzed. From the endophthalmitis database, information was obtained regarding the cause of endophthalmitis, microbiological work up including the vitreous isolates and their antibiotic susceptibilities, and the clinical outcomes. All patients with more than one isolate during microbiological workup were enrolled in the study.

The EVS recommendations were generally followed in postoperative endophthalmitis eyes. In endophthalmitis following open-globe injuries, three-port pars plana vitrectomy was performed in all eyes along with additional procedure if required (suturing scleral laceration, IOFB removal, endolaser, or silicone oil injection). Undiluted vitreous samples were sent immediately for microbiology evaluation. All eyes received intravitreal vancomycin (1.0 mg in 0.1 ml) and either amikacin (0.4 mg in 0.1 ml) or ceftazidime (2.25 mg in 0.1 ml) and additional intravitreal dexamethasone (0.4 mg in 0.1 ml) in postoperative cases. Intravitreal amphotericin B (5 μg in 0.1 ml) was administered on clinical suspicion based on surgeon’s treatment preference. Additional procedures were recorded when intravitreal antimicrobials or pars plana vitrectomy/vitreous lavage were repeated. Treatment and management decisions on secondary interventions were made by the individual treating physician without a predefined study protocol. Bacterial isolates were identified using Analytical Profile Index (API, bioMeriux, Marcy-l'Etoile, France). The antibiotic sensitivity was checked by Kirby Bauer disc diffusion method. Isolation of two or more different organisms from vitreous was considered to be a polymicrobial infection. The best-corrected visual acuity of less than 20/200 at final follow-up was defined as unfavorable visual outcome.

## Competing interests

The authors declare that they have no competing interests.

## Authors' contributions

AJ carried out the data analysis and drafted the manuscript. MRM and MK carried out the data collection. AP is one of the treating physician and also carried out the correction of the manuscript. SJ, AM, AM, TD are the other treating physicians. SS is the microbiologist. HWFJ corrected the manuscript. All authors read and approved the final manuscript.
